# Multifaceted Roles of cAMP Signaling in the Repair Process of Spinal Cord Injury and Related Combination Treatments

**DOI:** 10.3389/fnmol.2022.808510

**Published:** 2022-02-23

**Authors:** Gang Zhou, Zhiyan Wang, Shiyuan Han, Xiaokun Chen, Zhimin Li, Xianghui Hu, Yongning Li, Jun Gao

**Affiliations:** ^1^Department of Neurosurgery, Peking Union Medical College Hospital, Chinese Academy of Medical Sciences & Peking Union Medical College, Beijing, China; ^2^Plastic Surgery Hospital, Chinese Academy of Medical Sciences & Peking Union Medical College, Beijing, China; ^3^Department of International Medical Service, Peking Union Medical College Hospital, Chinese Academy of Medical Sciences & Peking Union Medical College, Beijing, China

**Keywords:** spinal cord injury, cAMP, multifaceted roles, combination treatments, cAMP compartmentation

## Abstract

Spinal cord injury (SCI) results in multiple pathophysiological processes, including blood–spinal cord barrier disruption, hemorrhage/ischemia, oxidative stress, neuroinflammation, scar formation, and demyelination. These responses eventually lead to severe tissue destruction and an inhibitory environment for neural regeneration.cAMP signaling is vital for neurite outgrowth and axonal guidance. Stimulating intracellular cAMP activity significantly promotes neuronal survival and axonal regrowth after SCI.However, neuronal cAMP levels in adult CNS are relatively low and will further decrease after injury. Targeting cAMP signaling has become a promising strategy for neural regeneration over the past two decades. Furthermore, studies have revealed that cAMP signaling is involved in the regulation of glial cell function in the microenvironment of SCI, including macrophages/microglia, reactive astrocytes, and oligodendrocytes. cAMP-elevating agents in the post-injury milieu increase the cAMP levels in both neurons and glial cells and facilitate injury repair through the interplay between neurons and glial cells and ultimately contribute to better morphological and functional outcomes. In recent years, combination treatments associated with cAMP signaling have been shown to exert synergistic effects on the recovery of SCI. Agents carried by nanoparticles exhibit increased water solubility and capacity to cross the blood–spinal cord barrier. Implanted bioscaffolds and injected hydrogels are potential carriers to release agents locally to avoid systemic side effects. Cell transplantation may provide permissive matrices to synergize with the cAMP-enhanced growth capacity of neurons. cAMP can also induce the oriented differentiation of transplanted neural stem/progenitor cells into neurons and increase the survival rate of cell grafts. Emerging progress focused on cAMP compartmentation provides researchers with new perspectives to understand the complexity of downstream signaling, which may facilitate the clinical translation of strategies targeting cAMP signaling for SCI repair.

## Background

Spinal cord injury (SCI) is a devastating neural trauma with an incidence of 54 patients per million people in the United States (Frontera and Mollett, 2017). Patients with SCI often live with serious sequelae, such as paralysis, bladder/bowel dysfunction, and chronic pain. The available clinical treatments for SCI include early surgical decompression, corticosteroid application, hypothermia or hyperbaric oxygen therapy, and rehabilitation training (Patel and Huang, 2017; Zhu, 2018; Karsy and Hawryluk, 2019). Although these methods partially alleviate injuries and promote functional recovery, their long-term effects remain limited. Currently, a wide range of treatments aimed at mitigating injury or promoting neural regeneration have brought SCI treatments to a new epoch (Hodgetts and Harvey, 2017; Zhang et al., 2018; Jin et al., 2019; Venkatesh et al., 2019). Among the novel strategies, manipulating cytoplasmic cAMP signaling has been proven to efficiently promote injury repair and functional recovery (Nikulina et al., 2004; Pearse et al., 2004). cAMP is an intracellular second messenger that can be activated by many stimuli and functions in key physiological processes to maintain cell homeostasis. Elevated neuronal cAMP levels not only enhance intrinsic axonal growth during the development process but also promote neural survival and regeneration after injury. Recently, cAMP has also been shown to regulate the function of glial cells and interact in complex ways with other signals generated by SCI, including macrophage/microglia polarization, astrocyte activation, and oligodendrocyte differentiation. However, therapies directly targeting cAMP are considered controversial because systemic elevation of this ubiquitously expressed molecule may cause dysfunction of other cells and additional deleterious effects. To address these concerns, researchers explored multiple approaches to expedite the clinical application of therapies involving cAMP signaling, such as targeting more specific downstream effectors and delivering agents in combination with biomaterials (Wang et al., 2011; Boczek and Kapiloff, 2020; Guijarro-Belmar et al., 2021). In this review, we highlight the research progress regarding the multifaceted roles of cAMP signaling in the repair process of SCI and focus on combination treatments that increase the bioavailability and specificity of cAMP-elevating agents, aiming to realize the therapeutic potential of cAMP in clinical settings.

## Pathophysiology of SCI

Based on its pathophysiology, SCI is classified into primary injury and secondary injury. The primary injury is caused by a mechanical force disrupting neural tissues and blood vessels. Following primary injury, the secondary injury includes multiple pathophysiological changes and is subdivided into the acute phase (<2 days), the subacute phase (2 days to 2 weeks), the intermediate phase (2 weeks to 6 months), and the chronic phase (>6 months) (Rowland et al., 2008). During the acute phase, continued tissue hemorrhage, ischemia, edema, and hypoxia result in a cascade of events including glutamate-mediated excitotoxicity, Ca^2+^ overload, and ROS production, leading to dysfunction and necrosis of neurons as well as oligodendrocytes. Simultaneously, peripheral immune cells infiltrate the lesion site and further exacerbate local inflammation due to the increased permeability of the blood-spinal cord barrier (BSCB; Kim et al., 2017). The subacute phase is characterized by the phagocytosis of inflammatory cells to clear debris and growth-inhibiting components from the lesion site (Rowland et al., 2008). Astrocytes are concomitantly activated to circumscribe the damage and form glial scars up to the end of the intermediate phase (Hill et al., 2001). However, the glial scars form a barrier for axonal regeneration both physically and chemically due to the expression of chondroitin sulfate proteoglycans (CSPGs). During the intermediate and chronic phases, the key events are tissue reconstruction including dynamic vascular remodeling, demyelination and remyelination, and reorganization of neural circuits (Badhiwala et al., 2018). The aforementioned stages are followed by a lifelong process of functional remodeling.

## Intracellular Camp Signaling Pathways

**Figure 1:**
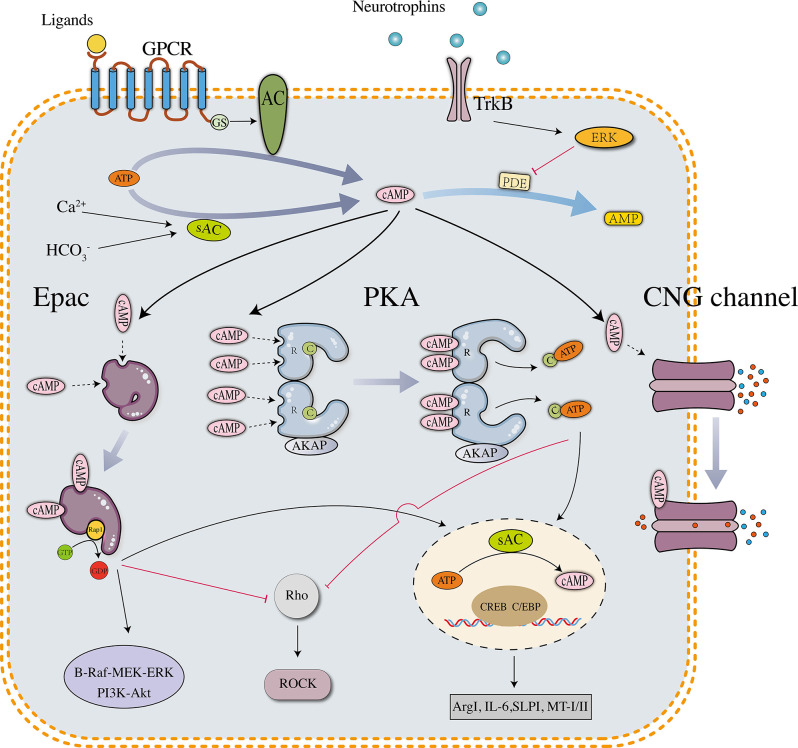
Downstream effectors and signaling pathways of neuronal cAMP. PKA, Epac, and CNG channels are the main downstream effectors of cAMP. The PKA holoenzyme has two regulatory (R) subunits and two catalytic (C) subunits. Each R subunit includes two cAMP-binding sites in the carboxy terminus. The C subunits are globular proteins that are responsible for ATP binding and peptide catalysis. When cAMP occupies the binding sites, the R subunits change their conformation and reduce the affinity with the C subunits. The PKA holoenzyme then dissociates into four subunits, and the disaggregated C subunits phosphorylate substrate proteins, such as the transcription factor cyclic AMP response element-binding protein (CREB). pCREB recruits CBP/P300, which has histone acetyltransferase (HAT) activity and facilitates gene transcription. In neurons, several downstream genes are involved in axonal regrowth, including arginase I (Arg I), secretory leukocyte protease inhibitor (SLPI), interleukin-6 (IL-6), and metallothionein I/II. Unlike PKA, which is a tetramer, Epac is a monomer in which the regulatory region sterically hinders the catalytic site to maintain an auto-inhibitory mode. The binding of cAMP induces a conformational change and promotes the removal of the regulatory lobe from the catalytic lobe, which exposes the GEF domain and allows the binding of Rap1. After binding Epac, Rap1 releases GDP to bind GTP and activates signaling cascades such as B-Raf-MEK-ERK and PI3K-Akt/PKB pathways. Activated PKA and Epac share similar downstream signaling pathways, including phosphorylating CREB and inhibiting the Rho-ROCK pathway. The tetrameric CNG channels anchored on the cell membrane are usually closed but undergo conformational changes after binding cAMP, leading to ion influx and biological reactions.

Intracellular cAMP is converted from ATP *via* a reaction catalyzed by adenylate cyclases (ACs), which include nine transmembrane members (tm-ACs, AC1–9) and one soluble member (sAC, AC10). tm-ACs are downstream effectors of G-protein coupled receptors (GPCRs) that are activated by extracellular molecules, AC1 and AC8 are also directly activated by Ca^2+^. sAC is activated by intracellular bicarbonate, Ca^2+^, and ATP (Kleinboelting et al., 2014). In neurons, sAC is the major source of cAMP that controls survival and growth signaling (Cameron and Kapiloff, 2017). cAMP is hydrolyzed by phosphodiesterases (PDEs), which include 11 subtypes in mammals. Different PDE subtypes have different affinities for cyclic nucleotides. PDEs 4, 7, and 8 are specific for cAMP, PDEs 5, 6, and 9 are specific for cGMP, and the other PDEs hydrolyze both cAMP and cGMP. In the mammalian central nervous system (CNS), the reported downstream effectors of cAMP include protein kinase A (PKA), exchange protein directly activated by cAMP (Epac), and cyclic nucleotide-gated channel (CNG channel; Robichaux and Cheng, 2018). PKA is expressed as two isoforms designated PKA (I) and PKA (II), which differ in their regulatory subunits (RI and RII) (Cheng et al., 2008). cAMP-PKA signaling has been proven to regulate physiological reactions in almost all cell types. Similar to PKA, Epac is also expressed as two isoforms, Epac1 and Epac2, which exhibit a different expression pattern in vertebrates. Epac1 is widely expressed in different tissues, whereas Epac2 is prominently expressed in neural tissues and the adrenal gland (Kawasaki et al., 1998). During the development of the mammalian spinal cord, Epac1 is expressed at high levels in the embryonic and neonatal stages but substantially downregulated in the adult stage. Epac2 is mainly expressed in the adult stage and accumulates in growth cones and neurites (Enserink et al., 2002; Zhou et al., 2016). Differences in the structural and developmental expression patterns between Epac1 and Epac2 may reveal their different functions: Epac1 expressed in early neurons contributes to primary axon development, and Epac2 expressed in mature neurons modulates the dendrite stability and outgrowth (Robichaux and Cheng, 2018). CNG channels were initially identified in photoreceptors and olfactory receptors, but evidence consistently proves their important functions in neurons and astrocytes (Kaupp and Seifert, 2002). The activation of CNG channels changes the levels of intracellular ions, which are related to neuronal excitability, synaptic plasticity, neurogenesis, and glial functions (Podda et al., 2012; Podda and Grassi, 2014). Although the three effectors are structurally different, they share an evolutionarily conserved sequence termed the cyclic nucleotide-binding (CNB) domain. cAMP binds to the CNB domain and activates downstream signaling pathways (details are shown in Figure 1).

## Multifaceted Roles of cAMP Signaling in The Repair Process of SCI

### Role of cAMP Signaling in Neuronal Survival and Axonal Regrowth

In adult mammals, CNS neurons do not regenerate after injury. One of the crucial reasons is that these neurons are ensheathed by inhibitory molecules such as Nogo-A, myelin-associated protein (MAG), and oligodendrocyte-myelin glycoprotein (OMgp) (Forgione and Fehlings, 2014). These molecules bind to the Nogo receptor (NgR) on the neuronal membrane and activate the Rho-ROCK pathway, which leads to growth cone collapse and limits neurite outgrowth (Fournier et al., 2001). However, these molecules are permissive to neurite outgrowth in the embryonic stage and become inhibitory during the course of development. For instance, P1 DRG neurons extend neurites when plated on top of cells expressing MAG, however, P5 DRG neurons do not extend neurites in the same condition (Cai et al., 2001). Because the endogenous cAMP level in DRG neurons is substantially decreased at P3–4 and remains at a low level in adults, this reverse effect indicates that intracellular cAMP might induce a switch between the growth-promoting or -inhibiting effects of myelin, as has been tested in multiple neuronal subtypes (Mukhopadhyay et al., 1994; Turnley and Bartlett, 1998; Cai et al., 2001). cAMP is also responsible for axonal guidance because low cAMP concentrations in the growth cone result in repulsion in the presence of guidance cues such as MAG and Netrin-1, but high concentrations result in attraction (Song and Poo, 1999). Elevating neuronal cAMP levels to promote axonal growth has been tested both *in vitro* and *in vivo*. Increasing cAMP levels through a conditioning lesion to DRG neurons enhances the regeneration of the central branch (Qiu et al., 2002). Similarly, the injection of db-cAMP into the lesioned dorsal column efficiently induces the axonal regrowth of DRG neurons (Neumann et al., 2002). In addition, adult DRG neurons plated on a myelin substrate with db-cAMP exhibit extensive neurite outgrowth, and the inhibitory effect of myelin is completely reversed. It is currently known that cAMP-induced neurite outgrowth has two phases, the first acting in the growth cone (transcription-independent phase) and the second in the cell body and nucleus (transcription-dependent phase) (Peace and Shewan, 2011). In the transcription-independent phase, elevated cAMP levels in the growth cone activate PKA and Epac, which can inhibit the Rho-ROCK pathway and enhance neurite outgrowth. The transcription-dependent phase is initiated by the activation of PKA and Epac in the cell body and subsequent phosphorylation of the cAMP response element-binding protein (CREB), which further promotes gene transcription involved in neurite outgrowth (Batty et al., 2017). Elevation of intracellular cAMP levels has also been shown to exert neuroprotective effects after SCI through multiple mechanisms, including enhancing the anterograde and retrograde transport of axons to retard diffuse axonal damage, phosphorylating Ca^2+^ channels and preventing Ca^2+^ overload, and activating the antiapoptotic MAPK-ERK pathway (Pearse et al., 2004). In addition, cAMP elevation potentiates the protective effects of neurotrophins by recruiting TrkB to the membrane from the cytoplasm (Meyer-Franke et al., 1998). In turn, neurotrophins-mediated activation of the TrkB-ERK pathway produces a transient inhibition of PDE4, leading to increased intracellular cAMP levels (Hannila and Filbin, 2008).

After the confirmation that cAMP plays a pivotal role in promoting neurite outgrowth and axonal guidance, it is a logical next step to identify downstream signaling pathways and underlying mechanisms. Early studies mainly focused on PKA, and the results proved its critical role. However, with the elucidation of the function of Epac, the effects on axonal regeneration that were previously attributed to PKA alone, have also been proved to be induced by Epac. In DRG neurons, the activation of Epac promotes extensive neurite outgrowth. Epac knockdown by siRNA significantly reduces neurite outgrowth and inhibits the growth-promoting effect of cAMP (Murray and Shewan, 2008). The activation of Epac2 reverses the inhibitory postlesion environment and promotes robust axonal outgrowth (Guijarro-Belmar et al., 2019). The relationship between the two effectors in axonal growth is now deemed complementary (Wei et al., 2016). When one pathway is inhibited, the other compensates to phosphorylate CREB and induce gene expression. In contrast, Epac and PKA exert a concentration-dependent antagonistic effect on growth cone chemotaxis: Epac promotes attraction to the guidance cue, whereas PKA promotes repulsion (Murray et al., 2009). In the presence of a high cAMP concentration, which is typically observed in embryonic neurons, cAMP activates both Epac and PKA. In this case, the attraction caused by Epac counteracts the repulsion caused by PKA. In the presence of a low cAMP concentration, which is typically found in adult neurons, cAMP only activates PKA and promotes growth cone repulsion (Batty et al., 2017). However, the complementarity or antagonism between Epac and PKA involves complex mechanisms that remain unclear. The net cellular effects of cAMP may not be generated by one effector alone but rather by the sum of all relevant pathways (Cheng et al., 2008). Clarifying the basis of crosstalk between downstream signaling pathways represents a novel direction for future research.

### Role of cAMP Signaling in Macrophage/Microglial Polarization

Macrophage/microglial polarization is a key event in secondary injury. The accumulation of macrophages/microglia in the lesion epicenter plays an important role in the inflammatory response during secondary injury. Macrophages/microglia are divided into two subtypes: M1 macrophages/microglia promote inflammatory reactions and tissue damage, whereas M2 macrophages/microglia promote anti-inflammatory reactions and tissue repair (Martinez et al., 2009; Gensel and Zhang, 2015). Depending on the polarized status and function, the phenotypic switch of macrophages/microglia from M1 to M2 is a novel therapeutic target in SCI repair (Kong and Gao, 2017).

Elevated intracellular cAMP levels in macrophages exhibit immunosuppressive activity predominantly by antagonizing proinflammatory cytokine production and phagocytosis (Serezani et al., 2008). However, proinflammatory cytokines such as TNF-α and IL1-β reduce cAMP levels in microglia by increasing PDE4 activity after SCI (Ghosh et al., 2012). These considerations lead to the hypothesis that cAMP elevation may attenuate the secondary injury caused by local inflammation. Ghosh et al. performed additional studies and subsequently showed that the concurrent application of db-cAMP and IL-4 promotes the polarization of microglia from the M1 to M2a phenotype *in vitro*. The underlying mechanism is that cAMP collaborates with IL-4 to activate PKA and the transcription factor CCAAT/enhancer-binding protein (C/EBP) and thereby induce Arg-1 (an M2 macrophage biomarker) expression (Sheldon et al., 2013; Ghosh et al., 2016). In SCI models, the administration of db-cAMP in combination with IL-4 facilitates the phenotypic change from M1 to M2a in both microglia and macrophage populations (Ghosh et al., 2016). Similarly, an injured spinal cord treated with roflumilast (a PDE4 inhibitor) exhibits not only significant reductions in the M1/M2 ratio and levels of pro-inflammatory molecules but also decreased cavity formation compared with the controls (Moradi et al., 2020). Recently, Negreiros-Lima further proved that cAMP regulates the major functions of macrophages through PKA, including nonphlogistic recruitment, phenotype switching, and efferocytosis, which are all pivotal processes in inflammation resolution (Negreiros-Lima et al., 2020).

Although cAMP promotes the polarization of macrophages/microglia and alleviates the inflammatory response, the intracellular mechanism remains unclear. cAMP is traditionally presumed to inhibit the activity of the transcription factor NF-κB to inhibit inflammatory gene expression. However, previous studies have also indicated that cAMP enhances NF-κB activity through its downstream effector PKA or Epac in macrophages (Gerlo et al., 2011). The network underlying the interaction between cAMP and other signaling pathways, such as MAPK and NF-κB, in macrophages and microglia remains to be further elucidated (Ghosh et al., 2015). Investigators have also recognized that macrophages do not form stable subsets but respond to a combination of factors in the microenvironment, and the polarization status is a continuum between M1 and M2 (Martinez and Gordon, 2014). A better understanding of the underlying mechanisms will enable researchers to promote the phenotype switch through cAMP signaling more efficiently.

### Role of cAMP Signaling in Astrocyte Activation

Astrocytes exhibit a two-step phenotypic change after SCI: naïve astrocytes (NAs) are activated to become reactive astrocytes (RAs) that exhibit cellular hypertrophy and extended processes. RAs subsequently migrate to the periphery of the lesion site, express cell adhesion molecules, and ultimately transform into scar-forming astrocytes (SAs) that adhere to each other to form the glial scars (Okada et al., 2018). Recent studies have confirmed that RAs are divided into two subsets: A1 astrocytes secrete neurotoxins that induce the death of neurons and oligodendrocytes, whereas A2 astrocytes secrete growth factors and promote neuroregeneration (Hassanzadeh et al., 2021). The key regulator of RAs in the repair process after SCI is the STAT3 signaling pathway, which is activated by cAMP and exerts neuroprotective effects by IL-6 (Okada et al., 2006; Tanabe et al., 2016). A global transcriptome analysis has revealed that increased cAMP levels in astrocytes can upregulate the expression of antioxidant-related genes and downregulate the expression of cell death-related genes (Paco et al., 2016). When activated by LPS *in vitro*, astrocytes simultaneously treated with an Epac2 agonist (S220) tend to exhibit lower GFAP expression and a preserved nonreactive morphology with slender processes, indicating that the increase in Epac2 activity might attenuate the activation of astrocytes. As observed in *ex vivo* SCI models, S-220-treated spinal cords show a closer relationship between neurite profiles and astrocyte processes, whereas nontreated spinal cords exhibit collapsed growth cones when confronted with RAs (Guijarro-Belmar et al., 2019). These results collectively indicate that cAMP signaling is a key regulator of RAs function after SCI. Recently, astrocytic scars have been reported to play a beneficial role in axonal regrowth. The ablation of chronic astrocytic scars fails to result in the regrowth of fibers, while scar-forming astrocytes permit and support robust axon regeneration in response to appropriate stimuli (Anderson et al., 2016). These results challenge the dogma that glial scars are detrimental to axonal regrowth. Scar-forming astrocytes upregulate and express specific CSPGs that support axonal regrowth. However, research on whether cAMP signaling is related to growth supporting effects is still lacking, which requires further investigation.

### Role of cAMP Signaling in Demyelination and Remyelination

Myelination is critical to maintain the integrity of the axonal structure. Proteins expressed in myelin help form nodes of Ranvier, which are essential for the saltatory conduction of action potentials. Myelin also alters the axonal cytoskeleton and provides metabolic support for neurons. The demyelination of axons after SCI not only subverts action potential conduction but also impairs the function and sustainability of bare axons (Pukos et al., 2019). Moreover, the regenerated axons in the injured spinal cord typically fail to become myelinated and their function is not restored (Pukos et al., 2019). Strategies targeting alleviating demyelination and promoting remyelination have therefore become options for injury repair. Because cAMP signaling promotes oligodendrocyte differentiation by inducing the expression of myelin enzymes and myelin sheath proteins during oligodendrocyte development, treatments targeting this pathway might be a method for promoting myelination after SCI (Malone et al., 2013). Previous studies have suggested that rolipram attenuates oligodendrocyte apoptosis and increases the number of oligodendrocyte-myelinated axons in the ventrolateral funiculus (VLF), which leads to improvements in the conductivity of descending and ascending axons and better hindlimb functional recovery in contusive SCI models (Whitaker et al., 2008; Beaumont et al., 2009). Evidence from demyelinating models suggests that cAMP promotes the maturation and differentiation of oligodendrocyte precursor cells (OPCs) through the activation of the MEK-ERK-MAPK pathway, leading to remyelination of axons (Sun et al., 2012; Syed et al., 2013).

### cAMP Signaling May Regulate the Repair Progress by the Interplay Between Neurons and Glia Cells in the Microenvironment After SCI

**Figure 2:**
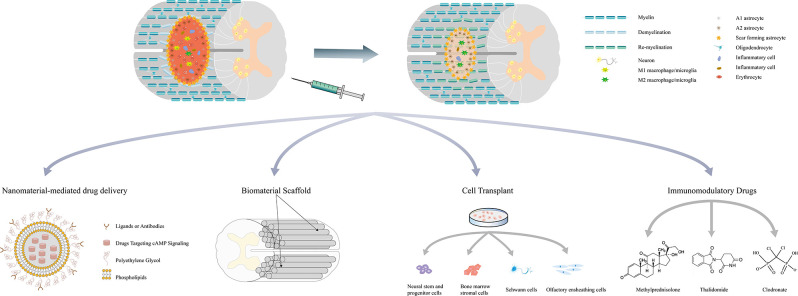
Multifaceted roles of cAMP signaling in the repair process of spinal cord injury and related combination treatments. Drugs targeting cAMP signaling delivered by nanoparticles and bioscaffolds can be released locally and avoid the systematic side effects. These drugs have also been combined with stem cell transplantation to promote survival and induce the differentiation of stem cells. Simultaneous application with other immunomodulatory drugs may regulate the immune response and mitigate secondary injury. Combination treatments can exert synergistic effects on attenuating inflammation, reducing cell death, minimizing the size of the lesion cavity, inhibiting CSPGs expression, promoting axon regeneration, and eventually leading to better functional recovery.

The literature review described above revealed that cAMP-elevating agents increase the cAMP levels in both neurons and glial cells and exert protective effects to promote tissue repair after SCI. During the acute and subacute phase of SCI, cAMP regulates the functions of macrophages/microglia and RAs to attenuate local inflammation, reduce the apoptosis of neurons and oligodendrocytes, and inhibit glial scar formation and demyelination. During the intermediate and chronic phases, activated cAMP signaling enhances axonal regeneration and remyelination (Figure 2). Previous studies have shown that the pathophysiology of SCI is a dynamic process that comprises multiple interactions between neurons and glial cells. For example, M1 macrophage-secreted factors induce a gene expression pattern of RAs, whereas factors from M2 macrophages inhibit the expression of these genes. Astrocytes stimulated by M2 macrophages in turn suppress both M1 and M2 macrophage proliferation and decrease TNF-α production in M1 macrophages (Haan et al., 2015). M1 macrophages/microglia secrete inflammatory molecules that contribute to the death of oligodendrocyte cells and lead to demyelination, whereas the myelin debris generated in the injured spinal cord modulates macrophage activation (Kong and Gao, 2017). Action potential propagation in axons fosters activity-dependent oligodendrocyte development and myelination through cAMP signaling (Malone et al., 2013). These interactions indicate that cAMP signaling may regulate injury repair through the interplay among cells in the post-injury environment. Increased cAMP levels in a certain cell not only change its own functional state but also subsequently exert protective effects on other cells and lead to a synergistic outcome. As numerous studies have confirmed that the administration of agents targeting cAMP signaling significantly promotes tissue repair and improves the outcome of SCI, future research should further elucidate the net effect of activating cAMP signaling in a certain cell *in vitro*, as well as focus on the exploration of novel approaches to refine treatments targeting specific cells.

## Combination Treatments Associated with cAMP Signaling in SCI

Given the protective role of cAMP signaling in the pathophysiology of SCI, treatments targeting cAMP signaling have become a potential strategy for SCI repair. As systemic increases in cAMP levels may have additional deleterious effects, more specific approaches are needed to improve the efficiency and feasibility of these agents in clinical settings. Currently, combination treatments associated with cAMP signaling are considered to have a bright future for SCI. Table 1 summarizes the combination treatments associated with cAMP signaling and most of the results show that the combinations exert better therapeutic effects than cAMP-elevating agents alone. One of the methodologies involves the incorporation of immunomodulatory drugs, including methylprednisolone, clodronate, and thalidomide (Koopmans et al., 2009; Iannotti et al., 2011; Yin et al., 2013). Methylprednisolone (MP) is by far the most generally accepted drug due to its significant effect on relieving secondary injury. A previous study showed that the treatment of rolipram in combination with MP in rat SCI models inhibits the expression of CSPGs, reduces motor neuron death, minimizes the size of the lesion cavity, increases the regeneration of corticospinal tract (CST) fibers, and ultimately enhances functional recovery after injury (Yin et al., 2013). *In vitro* evidence shows that rolipram and MP also upregulate MMP-2 expression in neurons and RAs, which facilitates CSPGs degradation and provides a more permissive environment for axon regeneration (Yin et al., 2013). However, these treatments could not avoid the systemic side effects of increased cAMP levels.

**Table 1:** Summary of combination treatments related to cAMP signaling in SCI.

SCI models	Combination treatments	Conclusions	References
Contusion(rat)	1. Rolipram and thalidomide are injected intraperitoneally soon after injury.	1. The combined application of rolipram and thalidomide leads to significantly improved locomotor performance, lower levels of IL-1β and TNF-α, and a greater degree of white matter sparing compared with the results obtained with rolipram or thalidomide alone.	Koopmans et al. (2009)
Contusion (rat)	1. Liposomal clodronate is injected intravenously immediately after injury and on postinjury days 1, 3, and 6. 2. Rolipram is delivered by subcutaneously implanted mini-osmotic pumps for 2 weeks starting immediately after injury.	1. The delivery of liposomal clodronate or rolipram alone promotes neuroprotection, increased myelinated tissue sparing, and improved locomotive recovery, and the combination treatment achieves the greatest effect.	Iannotti et al. (2011)
Hemisection (rat)	1. Liposomal clodronate is injected intravenously on postinjury days 1, 3, and 6. 2. Rolipram is delivered by subcutaneously implanted mini-osmotic pumps for 1 week starting immediately after injury. 3. Chondroitinase ABC (ChABC) is microinjected into the lesion center and cord stump 2 mm rostral and caudal to the lesion.	1. The combined treatment inhibits macrophage accumulation at the lesion site, reduces axonal retraction, diminishes the lesion size and cystic cavitation, and significantly improves locomotor function compared with the results obtained with the single treatments. However, the combined treatment does not induce substantial axonal regeneration throughout the lesion site.	Grosso et al. (2014)
Contusion and hemisection (rat)	1. Methylprednisolone (MP) sodium succinate is injected immediately after SCI. 2. Rolipram is delivered through a mini-osmotic pump for 14 days.	1. The combined treatment with rolipram and MP suppresses CSPGs expression, increases CSPGs degradation, reduces neuronal death, minimizes the size of the lesion cavity, increases the regeneration of the corticospinal tract (CST), and enhances functional recovery after injury.	Yin et al. (2013)
Hemisection (rat)	1. db-cAMP-loaded poly(propylene carbonate) (PPC) electrospun fiber sheets are placed on the hemisected spinal cord gap.	1. The encapsulation of db-cAMP in fibers leads to a stable and prolonged release *in vitro*. 2. The sustained delivery of db-cAMP promotes axonal regeneration and functional recovery and reduces glial scar formation.	Xia et al. (2013)
Transection (rat)	1. A scaffold functionalized by neutralizing proteins and collagen-binding neurotrophic factors is placed on the transected spinal cord gap. 2. db-cAMP is injected into the lesion site along with the functionalized collagen scaffold.	1. The functionalized collagen scaffold promotes neurite outgrowth in the presence of myelin. 2. The combination treatment shows greater advantages in reducing the cyst volume, facilitating axonal regeneration, and enhancing locomotion recovery than the single-method treatments.	Li et al. (2016)
Hemisection (rat)	1. db-cAMP- and ChABC-loaded poly (propylene carbonate) (PPC) electrospun fiber sheets are placed on the hemisected spinal cord gap.	1. The encapsulation of db-cAMP and ChABC in PPC fibers leads to the stable and prolonged release of each agent *in vitro*, and PPC electrospun microfibers are suitable for multidrug delivery. 2. The sustained delivery of db-cAMP and ChABC promotes axonal regeneration and functional recovery and reduces glial scar formation.	Xia et al. (2017)
Contusion (rat)	1. The S-220-loaded Fmoc hydrogel is injected into the dura in the contusion lesion area.	1. S-220 delivered by Fmoc hydrogels shows a relatively long pharmacological release profile, which is suitable for SCI repair. 2. S-220 delivered by Fmoc hydrogels significantly promotes neurite outgrowth and functional recovery in contusion SCI models.	Guijarro-Belmar et al. (2019)
Compression (rat)	1. Rolipram is encapsulated in poly(lactide-co-glycolide)-graft polyethyleneimine (PgP) and injected into the lesion site.	1. PgP increases the water solubility of hydrophobic drugs and sustainably releases the drugs. 2. Rolipram-loaded PgP (Rm-PgP) increases the intracellular cAMP level and promotes neurite outgrowth *in vitro*. 3. The injection of Rm-PgP into the lesion site reduces neuronal apoptosis and alleviates the inflammatory response.	Macks et al. (2018)
Compression (mice)	1. Rolipram is loaded into the selective and astrocyte-targeted nanogel (NG) (polyethylene glycol (PEG) and polyethylene-imine (PEI)), and the PEG-PEI NG is injected into the lesion site.	1. The PEG-PEI NG is exclusively internalized in astrocytes through the clathrin-dependent endocytic pathway. 2. Rolipram-loaded PEG-PEI NG reduces the production of inflammatory molecules in activated astrocytes and reverses the toxicity in motor neurons.	Vismara et al. (2020)
Hemisection (rat)	1. Drug-eluting microfibrous patches loaded with a low or high dose of rolipram are implanted subdurally following the injury to directly deliver rolipram to the spinal cord.	1. Rats treated with low-dose rolipram-loaded patches exhibit the greatest functional and anatomical recovery, the greatest degree of axon outgrowth, a marked increase in the number of oligodendrocyte cell populations (MBP+), and a decrease in the number of astrocytes compared with the results obtained from all other groups. 2. High-dose rolipram-loaded patches exert no significant effect on functional recovery and reduced survival rates due to systemic toxicity.	Downing et al. (2012)
Contusion (rat)	1. Rolipram is delivered by subcutaneously implanted minipumps for 2 weeks. 2. Schwann cells and db-cAMP are injected into the contused area.	1. Rolipram combined with Schwann cell grafts and db-cAMP injections increase the cAMP levels, promote axonal sparing and myelination, enhance the growth of serotonergic fibers into and beyond the grafts, and significantly improve locomotor functions.	Pearse et al. (2004)
Transection (rat)	1. cAMP is injected into bilateral L4 DRGs 5 d before spinal cord injury at C4. 2. Autologous bone marrow stromal cell (MSC) suspensions combined with NT-3 are injected into the lesion site to provide cellular matrix support for axonal growth.	1. The combination treatment with cAMP and NT-3 promotes significant axonal growth beyond the lesion site, and this effect was not observed after treatment with cAMP or NT-3 alone.	Lu et al. (2004)
Contusion (rat)	1. Rolipram is delivered subcutaneously *via* a silicon tube connected to an osmotic pump. 2. Olfactory ensheathing cells (OECs) are microinjected into the lesion site.	1. The combination treatment does not promote rubrospinal regeneration through the lesion site. 2. The systemic increase in cAMP levels induced by rolipram results in a greater number of OECs, a higher axonal density within the graft, and improved motor performance.	Bretzner et al. (2010)
Contusion (rat)	1. Rolipram is delivered subcutaneously *via* an osmotic pump. 2. Glial restricted precursor (GRP) cells are transplanted into the lesion site combined with db-cAMP injection.	1. The administration of rolipram and db-cAMP may reduce the survival or proliferation of GRP cells and result in a reduced graft size following transplantation into the injured spinal cord. 2. cAMP may not always be an ideal complement to progenitor cell transplantation after SCI.	Nout et al. (2011)
Transection (rat)	1. db-cAMP-loaded poly(lactic-co-glycolic acid) (PLGA) microspheres are embedded within the OPF hydrogel scaffolds. 2. PLGA-OPF scaffolds are placed in the Schwann cell (SC) or mesenchymal stem cell (MSC) culture medium for 24 h before surgery and then implanted in the transected spinal cord gap.	1. The db-cAMP-loaded PLGA microspheres incorporate into the OPF scaffolds and prolong the release of the drug. 2. The sustained delivery of db-cAMP inhibits axonal regeneration in the presence of Schwann cells but rescues the MSC-induced inhibition of axonal regeneration. 3. The sustained delivery of db-cAMP reduces capillary formation in the presence of SCs and MSCs, and this effect is coupled with reduced cyst/scar formation and better functional recovery.	Rooney et al. (2011)
Transection (rat)	1. db-cAMP is encapsulated in PLGA microspheres and embedded within chitosan guidance channels. 2. Neural stem/progenitor cells (NSPCs) are transplanted into the lesion site.	1. db-cAMP significantly increases transplanted NSPCs survival and induce NSPCs to directly differentiate into neurons both *in vitro* and *in vivo*. 2. The combination treatment of db-cAMP pretreated NSPCs transplanted with chitosan channels leads to extensive axonal regeneration and improved functional recovery.	Kim et al. (2011)
Contusion (rat)	1. Rolipram is delivered by subcutaneous minipumps for 2 weeks. 2. Schwann cells and db-cAMP are injected into the contused area.	1. The combination treatment group does not exhibit improved recovery, but the group treated with Schwann cells exhibits enhanced recovery only in some of the outcome measures.	Sharp et al. (2012)
Hemisection/transection (rat)	1. cAMP is injected into the reticular motor nucleus of the pons to stimulate the endogenous growth of neurons. 2. BDNF-expressing MSCs are injected into the lesion site through a glass micropipette to provide permissive matrices and stimulate the distal growth of motor axons.	1. The combination treatment enhances motor axon regeneration beyond both C5 hemisection and T3 complete transection sites. 2. Despite the formation of synapses beyond the lesion and a higher BBB score, the treated subjects exhibit worsened motor function. Higher BBB scores are potentially attributed to hyperactivity of the local spinal circuitry within the spinal cord below the transection site, which is an effect of persistent BDNF expression.	Lu et al. (2012)
Contusion (rat)	1. Rolipram is delivered by subcutaneous minipumps for 4 weeks. 2. GFP-D15A-transduced SCs are injected into the injury epicenter 1 week after SCI.	1. Rolipram combined with D15A-transduced SCs enlarges the SC grafts, increases the numbers of serotonergic fibers and axons in the grafts, and improves the hindlimb function compared with the results obtained with every single treatment alone. 2. Rolipram plays a key role in enhancing white matter sparing and increasing the myelinated axon content in the SC graft.	Flora et al. (2013)

Bioscaffolds are designed to provide mechanical support for axonal regeneration and serve as a local delivery system for agents that promote injury repair (Wang et al., 2011). However, the implantation of bioscaffolds may induce inflammatory responses that exacerbate the injury. Because cAMP exerts immunosuppressive effects on inflammatory cells, the combination of cAMP-elevating agents and bioscaffolds may not only specifically deliver drugs to the lesion site but also attenuate local inflammation. Previous studies have shown that db-cAMP delivered by poly (propylene carbonate) microfibers and collagen scaffolds released to the lesion site sustainably and significantly promotes injury repair (Xia et al., 2013, 2017; Li et al., 2016). Hydrogels have microporous structures that closely match the extracellular matrix of the spinal cord, and drugs loaded in hydrogels release more efficiently and durably at the lesion site. In an *ex vivo* SCI model, the injection of hydrogels loaded with S220, an Epac2 agonist, induces the bridging of axons across the lesion gaps in the organotypic spinal cord slices and enhances tissue repair (Guijarro-Belmar et al., 2019). Nanoparticles have been utilized to serve as vehicles to increase the delivery efficiency and bioavailability of drugs. Drugs delivered by nanoparticles coated with hydrophilic polymers such as polyethylene glycol (PEG) exhibit increased water solubility and exert similar or even better therapeutic effects at lower doses (Song et al., 2019). The third-generation of nanovectors can even target specific cells through the surface conjugation of a membrane recognition ligand (Boisseau and Loubaton, 2011). Vismara et al. developed a selective and astrocyte-targeted pharmacological delivery tool based on nanogels (polyethylene glycol (PEG) and polyethyleneimine (PEI)) loaded with rolipram. The nanogel is internalized exclusively in astrocytes through the clathrin-dependent endocytic pathway and it induces lysosome-oriented degradation and pharmacological release in mouse SCI models. The internalized rolipram-loaded nanogel reduces the production of inflammatory molecules, such as iNOS and Lcn2, in activated astrocytes and reverses toxicity in motor neurons *in vitro* (Vismara et al., 2020).

Drugs targeting cAMP signaling have also been combined with cell transplantation in SCI treatments. The candidate cell types include Schwann cells (SCs), neural stem and progenitor cells (NSPCs), bone marrow stromal cells (MSCs), and olfactory ensheathing cells (OECs). These cell grafts provide permissive matrices to synergize with the cAMP-induced inherent growth capacity of neurons. Researchers previously injected Schwann cells and db-cAMP into the lesion site of SCI models and delivered rolipram subcutaneously for 2 weeks. The combination treatment significantly promoted axonal regeneration growth and myelination, leading to improved motor function recovery (Pearse et al., 2004). Although these results were challenged by a subsequent study, the explanation for these opposite outcomes was attributed to differences in experimental procedures (Bunge and Pearse, 2012; Sharp et al., 2012). This synergistic effect of cAMP on cell grafts was also observed in OECs and bone MSCs (Lu et al., 2004; Bretzner et al., 2010). Activation of cAMP signaling in neural stem/progenitor cells (NSPCs) has been proven to induce the oriented differentiation of neurons (Zahir et al., 2009; Kim et al., 2011). NSPCs pre-treated with db-cAMP tend to exhibit not only a high survival rate but also more βIII-tubulin-positive neurons when transplanted into the injured spinal cord than untreated NSPCs (Kim et al., 2011).

## A Novel Direction of cAMP Signaling: cAMP Compartmentation

In the classic mammalian model, extracellular stimuli bind to GPCRs on the cell membrane and initiate a cascade of biochemical reactions, including the activation of tmACs, which catalyze the conversion of ATP to cAMP. cAMP then diffuses throughout the cell and activates its downstream effectors. However, this model could not explain how a single molecule efficiently responds to multiple extracellular stimuli or even how the same extracellular stimulus may result in the activation of different cAMP signaling pathways in different cellular compartments. After decades of investigation, it is currently believed that intracellular cAMP signaling is precisely compartmentalized by spatial and temporal regulation through the generation and catabolism of cAMP, which restricts cAMP diffusion to subcellular domains such as the cytoplasm, mitochondria, endoplasmic reticulum, and nucleus (Arora et al., 2013; Zaccolo et al., 2021). The contributors to cAMP compartmentalization include a large family of molecules involved in cAMP signaling, including GPCRs, ACs, PKA, Epac, PDEs, and A kinase anchoring proteins (AKAPs) (Zaccolo et al., 2021). AKAPs bind to the R subunits and tether PKA holoenzyme to specific subcellular locations in physical proximity to PKA targets to form a signalosome, thus increasing the specificity and efficiency of signaling (Robichaux and Cheng, 2018). sAC also facilitates cAMP compartmentalization to achieve a localized activity in cellular compartments, and thus the activation of downstream signaling does not require the long-distance diffusion of cAMP from the cell membrane (Stiles et al., 2014). Currently, intracellular cAMP signalosomes have been identified in the plasma membrane, the mitochondria, the Golgi apparatus, the endoplasmic reticulum, and the nucleus (Zaccolo et al., 2021). For example, cAMP compartmentalization in the nucleus is attributed to AKAP8, which anchors PKA and PDE4 in the nuclear matrix and forms a signalosome that is involved in CREB phosphorylation (Zaccolo et al., 2021). When cAMP is produced by the nuclear sAC (AC10) and reaches the threshold level, the PKA holoenzyme in the signalosome will be activated, leading to CREB phosphorylation and increased gene transcription (Sample et al., 2012). The discovery of nuclear sAC modified the conventional view that nuclear cAMP-PKA signaling depends on the activation of the cytosolic PKA holoenzyme and the diffusion of C subunits into the nucleus. In contrast, the nuclear sAC signaling cascade promotes the activation of CREB without requiring the translocation of any components, leading to a rapid response to changes in intrinsic signals (Zippin et al., 2004).

Neuronal cAMP signaling is involved in cell metabolism, axon extension, growth cone guidance, neuroprotection, and survival. The complex functional networks in neurons depend on cAMP compartmentalization: sAC activation increases intracellular cAMP levels and further activates PKA by dissociating the R and C subunits. AKAP scaffolds control cAMP signals specifically by inducing the PKA-mediated phosphorylation of downstream effectors such as ERKs, which promote survival and growth signaling. cAMP also specifies pro-survival signaling and gene expression *via* nuclear sAC-mediated compartmentalized synthesis (Cameron and Kapiloff, 2017). Neuronal sAC is necessary to increase CREB phosphorylation and regulate the transcription of genes required for cell survival and axon growth after injury (Corredor et al., 2012). An in-depth understanding of cAMP compartmentation offers researchers the opportunity to develop molecules that target cAMP domains rather than global intracellular cAMP levels. For example, Boczek et al. identified a novel perinuclear neuronal cAMP compartment that is related to neuronal protection and axonal growth. This compartment is organized by the scaffold protein mAKAPα (AKAP6α), and the activation of mAKAPα-associated cAMP signaling increases retinal ganglion cell survival after injury both *in vitro* and *in vivo* (Boczek et al., 2019). In addition, AKAP5 (AKAP150) is a key regulator that balances the Epac- and PKA-mediated phosphorylation of protein kinase B (PKB/Akt), a signaling molecule involved in neuronal survival and axonal outgrowth (Nijholt et al., 2008). Modulation of AKAP5 activity may enhance the PKB/Akt signaling pathway and promote injury repair.

## Summary and Future Directions

The repair process of SCI is quite intricate due to the interactions between neurons and glial cells. Comprehensive strategies that target different pathological processes simultaneously may yield a better response with respect to injury inhibition and neural regeneration. In this review, we provide a systematic outline of the pivotal roles of cAMP signaling involved in the repair process of SCI. We also summarize the combination treatments related to cAMP signaling in experimental SCI models, and most of the studies reported inspiring outcomes. Future research may focus on the following areas to promote the clinical translation of strategies targeting cAMP signaling: (1) further investigating the roles of cAMP signaling in different cells, particularly glial cells, under physiological and pathological conditions; (2) promoting the entry of combination strategies into preclinical or clinical trials to further confirm their safety and efficacy; and (3) comprehensively elucidating cAMP compartmentalization to more precisely modulate spatiotemporal intracellular cAMP signaling. All of these efforts will promote the development of new therapeutic strategies for SCI and ultimately benefit the patients.

## Authors Contributions

GZ and JG wrote the manuscript. ZW drew the figures. SH and YL helped write the manuscript and reviewed the manuscript. All authors reviewed and approved the final manuscript. All authors contributed to the article and approved the submitted version.

## Conflict of Interest

The authors declare that the research was conducted in the absence of any commercial or financial relationships that could be construed as a potential conflict of interest.

## Publisher’s Note

All claims expressed in this article are solely those of the authors and do not necessarily represent those of their affiliated organizations, or those of the publisher, the editors and the reviewers. Any product that may be evaluated in this article, or claim that may be made by its manufacturer, is not guaranteed or endorsed by the publisher.
